# Differential Metabolic Profiles during the Albescent Stages of ‘Anji Baicha’ (*Camellia sinensis*)

**DOI:** 10.1371/journal.pone.0139996

**Published:** 2015-10-07

**Authors:** Chun-Fang Li, Ming-Zhe Yao, Chun-Lei Ma, Jian-Qiang Ma, Ji-Qiang Jin, Liang Chen

**Affiliations:** Key Laboratory of Tea Biology and Resources Utilization, Ministry of Agriculture, Tea Research Institute of the Chinese Academy of Agricultural Sciences, Hangzhou, China; University Paris South, FRANCE

## Abstract

‘Anji Baicha’ is an albino tea cultivar with white shoots at low air temperature and green shoots at high air temperature in early spring. The metabolite contents in the shoots dynamically vary with the color changes and with shoot development. To investigate the metabolomic variation during the albescent and re-greening stages, gas chromatography–mass spectrometry combined with multivariate analysis were applied to analyze the metabolite profiles in the different color stages during the development of 'Anji Baicha' leaves. The metabolite profiles of three albescent stages, including the yellow-green stage, the early albescent stage, and the late albescent stage, as well as the re-greening stage were distinguished using principal component analysis, revealing that the distinct developmental stages were likely responsible for the observed metabolic differences. Furthermore, a group classification and pairwise discrimination was revealed among the three albescent stages and re-greening stage by partial least squares discriminant analysis. A total of 65 differential metabolites were identified with a variable influence on projection greater than 1. The main differential metabolic pathways of the albescent stages compared with the re-greening stage included carbon fixation in photosynthetic organisms and the phenylpropanoid and flavonoid biosynthesis pathways. Compared with the re-greening stage, the carbohydrate and amino acid metabolic pathways were disturbed during the albescent stages. During the albescent stages, the sugar (fructofuranose), sugar derivative (glucose-1-phosphate) and epicatechin concentrations decreased, whereas the amino acid (mainly glycine, serine, tryptophan, citrulline, glutamine, proline, and valine) concentrations increased. These results reveal the changes in metabolic profiling that occur during the color changes associated with the development of the albino tea plant leaves.

## Introduction

Albescence, the loss of chlorophyll from plants, can be caused by a number of genetic and environmental factors. Two albino tea cultivars can be induced by low air temperature or high-light conditions. ‘Anji Baicha’ is an albino tea cultivar with new white shoots at low air temperatures; however, the plant restores its green shoots with an increase in air temperature [1]. Changes in leaf color mainly occur due to chloroplast development, and chlorophyll accumulation is inhibited at low air temperatures. When the air temperature is increased, the chloroplast structures and chlorophyll content recover, and the leaf color returns to green [1]. The tea made with ‘Anji Baicha’ has a light green color and brisk taste due to albescence. The metabolic changes during the albescent stage, such as high free amino acid and low purine alkaloid and caffeine contents, contribute to the brisk taste of this tea [2–4]. Chemical content and gene expression analysis of the different stages of albino cultivars have revealed the effect of albescence on the tea plant at the biochemical and molecular levels [1,3–5]. To assess the influence of albescence on global gene expression levels in the tea plant, Ma *et al*. used a cDNA microarray to analyze gene expression and found that genes involved in energy metabolism, carbon fixation, and secondary metabolism were significantly changed during five albescent stages, including the yellow-green stage, the slightly white stage, the pale-white stage, the slightly green stage and the light-green stage [6]. Proteomic analysis revealed that the differentially expressed proteins in three stages, including the pre-albinistic stage, the albinistic stage and the re-greening stage, were mainly involved in carbon, nitrogen and sulfur metabolism [7]. Compared with the green tea cultivar ‘Longjing 43’, higher theanine and free amino acid and lower carotenoid, catechin and anthocyanin concentrations were found in another chlorina tea plant cultivar, ‘Zhonghuang 2’. These differences in metabolite concentrations mainly resulted from differential gene expression in ‘Zhonghuang 2’ compared with ‘Longjing 43’ [8]. Based on these gene, protein and metabolite results [2–7], we predicted that the metabolic pathways of ‘Anji Baicha’ during albescent stages were significantly different than those during the normal green stage. Previous research in these albino cultivars revealed the chemical content and gene expression changes in tea plants from the yellow-green stage to the white stage until the re-greening stage. However, changes in the comprehensive metabolic profiles of albino tea cultivars must be assessed to further improve tea cultivars.

Global metabolite profiling analyses enable the monitoring of metabolites profiles changes in the samples. Such analyses can be used to detect and monitor metabolite changes that play key functions in physiology and metabolism. Recent advances in bioanalytical separation and detection technologies, combined with rapid progress in bioinformatics, have made it possible to measure much larger bodies of metabolite data and to screen changes in metabolites in plants [9–12]. Metabolomic analysis of the tea plant has been widely used to study geographical and climatic influences [13], shade and air temperature influences [14,15], plucking positions [16] and metabolome-based health-promoting attributes of diverse tea cultivars [17]. Metabolite profiling using gas chromatography coupled to mass spectrometry (GC–MS) can detect various metabolites such as sugars, amino acids, amines, organic acids, phosphorylated metabolites and small secondary metabolites [18]. To reveal the dynamic metabolite profile changes in the yellow-green (YG) stage, the early albescent (WI) stage, the late albescent (WII) stage, and the re-greening (G) stage of the leaves, metabolites were detected by GC–MS and assessed by multivariate analysis. The metabolites that could distinguish the YG, WI, WII, and G stages were identified using principal component analysis (PCA) followed by partial least squares discriminant analysis (PLS-DA). The metabolic pathways that involve the identified differential metabolites were also investigated. These results reveal the metabolic profiling changes that occur with color changes during the development of albino tea plant leaves.

## Materials and Methods

### Plant material

Tea plants [*Camellia sinensis* (L.) O. Kuntze cv. ‘*Anji Baicha*’] were grown in the China National Germplasm Hangzhou Tea Repository (CNGHTR) of the Tea Research Institute of the Chinese Academy of Agricultural Sciences (TRICAAS). In early spring, the seasonal low air temperatures produced pre-albescent shoots with yellow-green leaves. As the shoots developed, the first leaves became second leaves in the WI stage. These second leaves were slightly white. When the shoots reached the WII stage with three or four leaves in late spring, the second or third leaves were white. Subsequently, the color of the shoot leaves began to gradually turn green as the air temperature increased. The yellow-green leaves from the YG stage were the first leaves, harvested on April 1, 2014. The slightly white leaves from the WI stage were the second leaves, collected on April 9, 2014. The pale-white leaves from the WII stage were the third or fourth leaves, collected on April 15, 2014. The green leaves from the G stage were the fifth or sixth leaves, obtained on May 9, 2014. Each sample was separately harvested from eight individual tea plants to obtain eight biological replicates. All collected samples were immediately frozen in liquid nitrogen and stored at –80°C until analyzed. The air temperature of the harvested day was obtained from the weather service (www.tianqi.com).

### Chemicals

HPLC-grade methanol and acetonitrile were purchased from Merck Chemicals (Darmstadt, Germany). BSTFA (containing 1% TMCS) was purchased from REGIS Technologies (Morton Grove, IL, USA). Epicatechin, citrulline, glucose-1-phosphate, fructose, glutamine, glycine, alanine, proline, serine, tryptophan and valine were purchased from Sigma-Aldrich (St. Louis, MO, USA).

### Chlorophyll measurement

Chlorophyll was extracted with 80% acetone from 100 mg of fresh leaf samples. The extract was measured spectrophotometrically at 645 and 663 nm. The chlorophyll content was quantified according to the method of Arnon [19] in five independent biological replicates. Statistical analysis of data was performed using Student's t test for comparisons of the chlorophyll in YG, WI, and WII with that in G.

### Metabolite extraction and data acquisition using GC–MS

Leaf samples were pre-cooled with liquid nitrogen and homogenized using a mortar and pestle. Then, 60 mg of powder was added to 360 μL of cold methanol and 40 μL of internal standard (0.3 mg mL^−1^ L-2-Cl-phenylalanine in methanol). The metabolite fractionation was completed as described by Wagner *et al*. [20]. After extraction, the samples were derived by the trimethylsilylation of acidic protons using 45 μL BSTFA, as previously described [21,22].

### Data acquisition by GC–TOF-MS

For each stage, eight biological replicates were independently analyzed. In total, 32 samples were randomly analyzed to reduce analysis bias. Chromatography was performed with a GC–TOF-MS Pegasus 4D (LECO, St. Joseph, MI, USA) with a DB–5 capillary column (30 m×250 μm inner diameter (i.d.), 0.25-μm film thickness; Agilent J&W Scientific, Folsom, CA). A derivatized sample (1 μL) was injected in split-injection mode into the DB–5 capillary column with a split ratio of 30:1. Helium was used as the carrier gas at a flow rate of 1 mL/min. The temperature was raised to 90°C in the first 0.2 min, then raised from 90°C to 160°C for 7 min, from 160°C to 220°C for 12 min, and from 220°C to 280°C for 3 min, and then maintained at 280°C for 7 min. The injection and ion source temperatures were set to 280°C and 220°C, respectively. Electron impact ionization (70 eV) in full scan mode (m/z 30–600) at a rate of 100 scans/s was performed to collect data.

Quality control (QC) samples were prepared by mixing 80 mg of one leaf sample from each of the four groups to become a combined sample, which was then divided into four QC samples and analyzed using the same method as for the experimental samples. The QC samples were injected at regular intervals (every eight samples) throughout the analytical run to provide a set of data from which repeatability could be assessed [23].

### Data processing and compound identification

All chromatograms were processed using ChromaTOF software (v 4.34, LECO, St. Joseph, MI). The peak list was exported as a CSV file containing sample information, retention times and raw spectra with absolute intensities. The internal standard was used for data quality control, which could reflect the reproducibility of the analysis. The noise elimination level was set at 10.00. Peaks caused by column bleed and BSTFA derivatization were identified using the negative control, which did not contain any leaf sample but was treated in the same manner as the other samples. Pseudo-positive peaks, such as peaks caused by column bleed and BSTFA derivatization were removed from the data set. The data set was normalized using the sum of the intensity of the peaks in each sample and equaled the sum of the intensity of the peaks in each sample to 1000. Metabolite annotation with the NIST 05 Standard mass spectral database and Fiehn database linked to the ChromaTOF software were manually checked with a similarity of more than 70%. When the results of the two databases were not consistent, the high degree of similarity of the metabolite name was chosen as the annotation result. Certain differential metabolites annotation results were validated by reference standards (S1 Table)

### Data analysis

The normalized data sets were analyzed using SIMCA-P+11.5 (Umetrics, Umea, Sweden) for multivariate statistical analysis. PCA was conducted to detect the intrinsic variation in the samples from different stages. PLS-DA was employed to maximize sample separation. Overfitting of the PLS-DA models was checked using a permutation test. Jackknife cross-validation was applied to validate the PLS-DA models [24]. PLS-DA was used to distinguish the differences in metabolic profiles between sample groups. A coefficient loading plot of the PLS-DA models was used to determine the differential metabolites that could separate the sample groups. R^2^ and Q^2^ values were used to verify the quality of the models. R^2^ reflects the goodness of fit and is defined as the proportion of variance in the data explained by the PLS-DA model. Q^2^ reflects predictability and is defined as the proportion of variance in the data predicted by the PLS-DA model [25]. The variable influence on projection (VIP) value of a metabolite greater than 1 demonstrated that it contributed greatly to the separation of sample groups in the PLS-DA models [26]. To prevent model overfitting, seven cycles of cross-validation and 200 repeats of response sequence validation were performed to examine the quality of the PLS-DA model. Variables responsible for distinguishing the three different albescent stages (YG, WI and WII) and the G stage were identified using the loading plots and VIP value thresholds (VIP > 1) obtained from the PLS-DA models. Then, metabolites for which the distribution of the concentrations and logarithmically transformed concentrations in this study had a normal distribution and homogenous variability were analyzed with Student’s t test (parametric tests); metabolites for which the distribution failed to meet these criteria were analyzed with Kruskal-Wallis (nonparametric tests). Only the metabolites with a p value < 0.05 were defined as differential metabolites.

To further interpret the biological significance associated with color changes of leaves, we used the Kyoto Encyclopedia of Genes and Genomes (KEGG) database to link differential metabolites to metabolic pathways in the YG, WI, WII stages compared with those in the G stage. Enrichment p-values were computed from a hypergeometric distribution. A p-value cutoff of 0.01 was selected to reduce the false discovery rate.

### Quantification of fructose in leaf samples by GC–TOF-MS

Five replicates of leaf samples in each stage were derivatized and subsequently analyzed by GC–TOF-MS according to the following method. A 0.15-g aliquot of the leaf sample was extracted with 0.7 mL methanol/water (3:1, v/v) and added 50 μL of L-2-chlorophenylalanine (0.1 mg/mL stock in dH_2_O) as an internal standard. The mixture was vortexed for 10 s and homogenized in a ball mill for 5 min at 55 Hz, followed by centrifuging at 12,000 rpm for 15 min at 4°C. An aliquot of the 400-μL supernatant was transferred into a glass sampling vial for vacuum-drying at room temperature for 1.5 h. The residue was derivatized using a two-step procedure. First, 80 μL methoxyamine (20 mg/mL in pyridine) was added to the vial, vortexed for 30 s and incubated at 80°C for 20 min in an oven, followed by the addition 100 μL BSTFA (containing 1% TCMS, v/v) into each sample and incubation at 70°C for one hour.

A 1-μL aliquot of the derivatized solution was injected in splitless mode into an Agilent 7890 gas chromatograph system coupled with a Pegasus HT time-of-flight mass spectrometer. The system utilized a DB-5MS capillary column coated with 5% diphenyl cross-linked with 95% dimethylpolysiloxane (30 m×250 μm i.d., 0.25-μm film thickness; J&W Scientific, Folsom, CA, USA). Helium was used as the carrier gas, the front inlet purge flow was 3 mL/min, and the gas flow rate through the column was 20 mL/min. The initial temperature was maintained at 50°C for 1 min, then raised to 330°C at a rate of 10°C/min, and then maintained for 5 min at 330°C. The injection, transfer-line, and ion-source temperatures were 280, 280, and 220°C, respectively. The energy was –70 eV in electron-impact mode. The mass spectrometry data were acquired in full-scan mode with an m/z range of 85–600 at a rate of 20 spectra per second after a solvent delay of 366 s.

Fructose standard was dissolved with methanol/water (3:1, v/v) to a final dilution of 100 μg/mL. The diluted standards were mixed and then derivatized and analyzed as the leaf samples, which produced the standard curves. The concentration of fructose was determined based on standard calibration curves. Statistical analysis was performed using Student's t test for comparison the fructose concentration in YG, WI, and WII with that in G.

### Quantification of differential metabolites in leaf samples by ultra performance liquid chromatography–triple quadrupole mass spectrometry (UPLC–QqQ-MS)

A 100-mg aliquot of leaf sample in each stage was ground for 3 min with a steel ball in 2-mL EP tubes, and then 500 μL methanol/water (3:1, v/v) was added to it. The mixture was centrifuged at 12,000 rpm for 10 min at 4°C, and the resulting supernatants were used for UPLC–QqQ-MS analysis. Five replicates of each sample were extracted and analyzed.

A 5-μL aliquot of the supernatant was injected into a column (100×2.0 mm, 3-μm NH_2_) (Phenomenex, Torrance, CA, USA) held at 40°C using an Agilent 6400 Triple Quadrupole LC/MS system (Agilent Technologies, Santa Clara, CA, USA). The mobile phase of the binary gradient elution consisted of (A) water and (B) acetonitrile, and separation was performed using the following gradient: 85% B from 0–2 min, 75% B from 2–9 min, 100% A from 9–15 min, and 85% B from 15–20 min. The flow rate was 0.4 mL/min. All samples were kept at 4°C during the analysis. Samples were analyzed by UPLC-QqQ-MS in the multiple reaction monitoring (MRM) mode to maximize sensitivity. The source temperature was set at 100°C, and a desolvation gas temperature of 300°C was used with a gas flow of 3.0 L/min. Data analysis was performed with Agilent Mass Hunter Qualitative Analysis B.04.00 Software (Agilent Technologies, Santa Clara, CA, USA).

Standards of epicatechin, citrulline, glucose-1-phosphate, glutamine, glycine, alanine, proline, serine, tryptophan and valine were dissolved with methanol/water (3:1, v/v) to a final dilution of 100 μg/mL. The diluted standards were mixed to produce the standard curves. The concentrations of epicatechin, citrulline, glucose-1-phosphate, glutamine, glycine, alanine, proline, serine, tryptophan and valine were determined based on standard calibration curves. Statistical analysis of data was performed using Student's t test for comparisons of the metabolites concentrations in YG, WI, and WII with those in G.

## Results and Discussion

### Color and chlorophyll concentration changes of tea leaves from the yellow-green stage to the white stage until the re-greening stage

**Fig 1:**
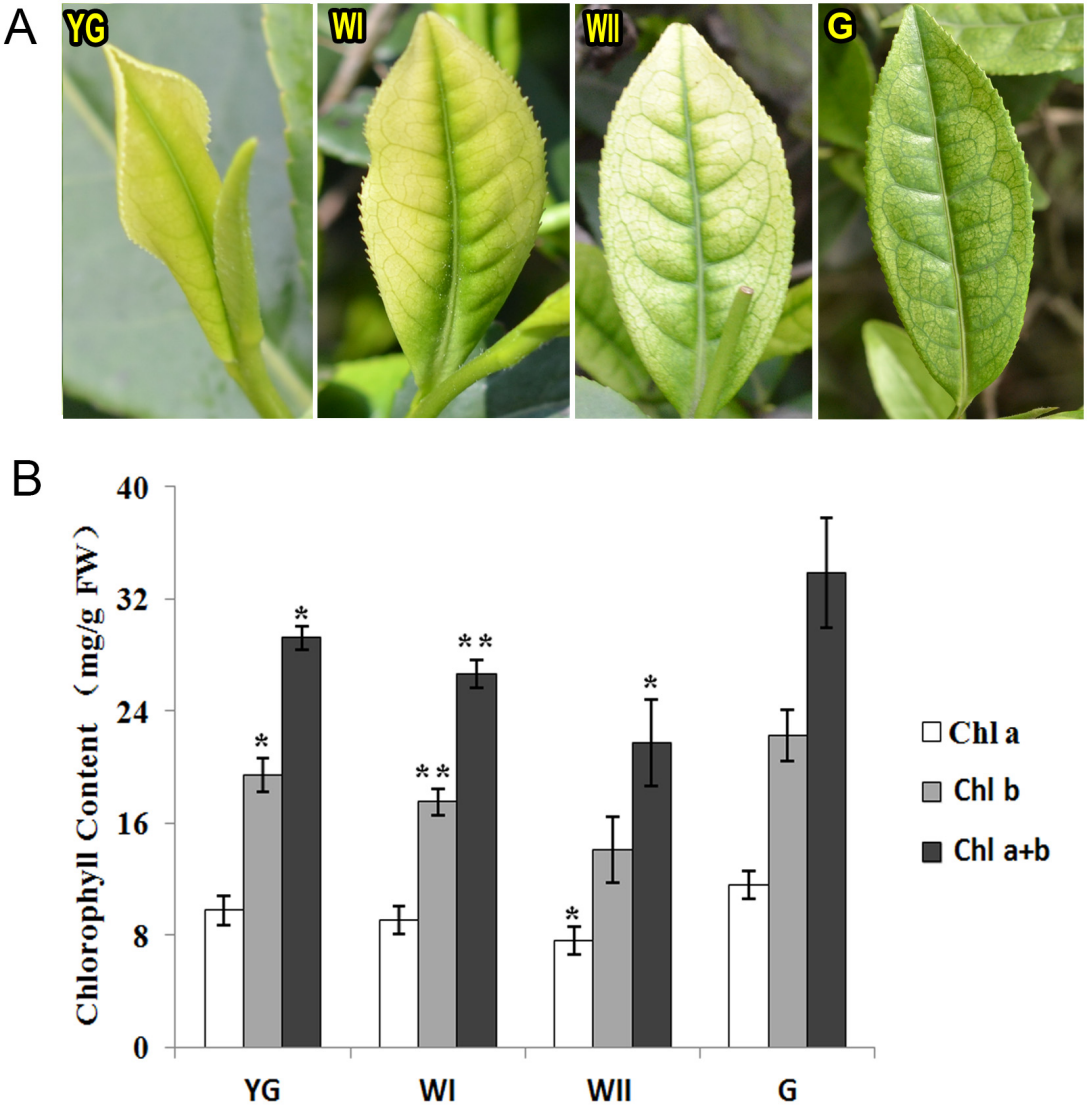
‘Anji Baicha’ leaves and chlorophyll concentration at four different stages. (A) ‘Anji Baicha’ leaves at four different stages during the development. YG, yellow-green leaf; WI, slightly white leaf; WII, pale-white leaf; G, re-greening leaf. (B) Chlorophyll a (Chl a), chlorophyll b (Chl b), and Chl a + b concentrations of ‘Anji Baicha’ leaves at four different stages. The significance of differences in the YG, WI and WII stages compared with that in the G stage is indicated with * (P < 0.05; Student's t test) or ** (P < 0.01; Student's t test).

The ‘Anji Baicha’ used in this research is an albino tea cultivar. The color of its new shoots is sensitive to the air temperature. In early spring, when the air temperature is less than 20°C, new shoots are yellow-green or yellow-white in color. As the air temperature increases, the new shoots gradually recover their green color. Based on the changes in leaf color as new shoots developed during the early spring, we divided the albescent process into three albescent stages, including the YG stage, the WI stage, and the WII stage, as well as the G stage (Fig 1A). The newly germinated, yellow-green single leaf and bud stage was defined as the YG stage. As this leaf and bud developed, the leaf became light green, and the edge of the leaf was yellow. This color pattern was defined as the WI stage. As the first leaf continued to develop, it became off-white, and only the leaf vein was green. This pattern was defined as the WII stage. When the air temperature exceeded 22°C, the leaves gradually turned green, similarly to other common tea cultivars; this color pattern was defined as the G stage. Table 1 showed the harvested time, air temperature and related stage in the previous research for each stage leaves. The chlorophyll a + b concentrations in the YG, WI and WII stage leaves were significantly lower than those in the G stage leaves (p < 0.05; Student's t test) (Fig 1B). Such reductions in the chlorophyll abundance might lead to color changes during leaf development from the albescent stage to the re-greening stage. Du *et al*. also found that the albino phenotype is strongly related to tea plant chlorophyll biosynthesis, which is inhibited at low air temperatures and recovers as the air temperature increases [1]. The reduced chlorophyll synthesis induces a complex perturbation of metabolite accumulation in the tea plant; for example, more amino acids and fewer polyphenols are found in albino leaves [3–5].

**Table 1:** Sample information and the related stage in the references.

Stage	Harvested time	Mean air temperature	Related stage in reference [6]	Related stage in reference [7]
YG	2014-04-01	14.0°C	Yellow-green shoot	Pre-albinistic stage
WI	2014-04-09	18.5°C	Slightly white shoot	—
WII	2014-04-15	17.0°C	Pale-white shoot	Albinistic stage
G	2014-05-09	21.0°C	Light-green shoot	Re-greening stage

### Principal component analysis (PCA) reveals dynamic changes in the metabolite profiles from the three albescent stages (YG, WI and WII) and the G stage

Metabolomics approaches can be used to characterize and quantify metabolites in the biological samples. To discover global metabolic profile changes during the three albescent stages (YG, WI and WII) and the G stage, GC–MS was applied to detect dynamic metabolite changes. For each stage, eight independent biological replicates were analyzed. After GC–MS analysis, mass spectral deconvolution and removal of the internal standard and pseudo-positive peaks, such as those caused by noise, column bleed and BSTFA derivatization, the number of peaks detected were 1023, 985, 997, and 981 in the YG, WI, WII, and G stages, respectively (S2 Table). Each peak was originally searched in the NIST and Fiehn databases to identify the metabolites. The relatedness of the four stages to each other was examined using PCA with the data presented in S3 Table. PCA can extract and display the systematic variation between the samples. A PCA model is the basis for metabolomic analysis, which provides a summary, or overview, of all observations or samples. Additionally, groupings, trends, and outliers can also be found by PCA [27].

**Fig 2:**
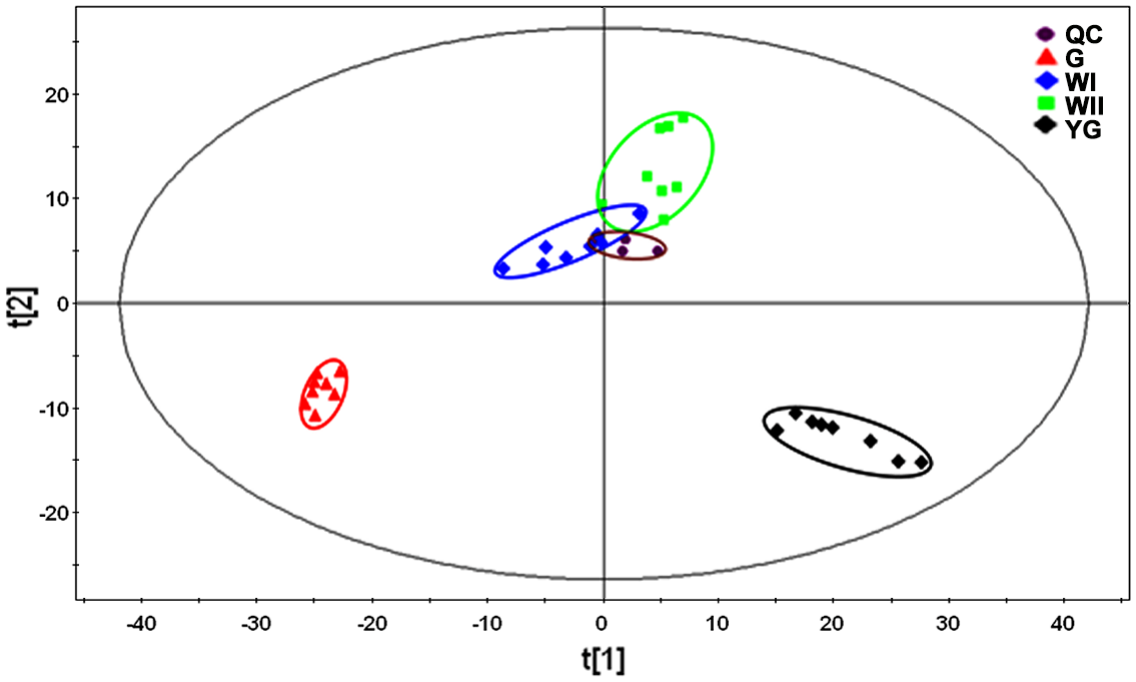
PCA was used to compare the metabolic profiles of the tea plant at three albescent stages (YG, WI and WII) and the G stage. Score plots for principle components 1 and 2 showed high cohesion within groups and good separation among leaf groups from the three different albescent stages (YG, WI and WII) and the G stage. The sampling groups are color coded as follows: black = YG-stage leaves; blue = WI-stage leaves; green = WII-stage leaves; red = G-stage leaves; and purple = QC samples.

The multi-dimensionality of complex data was reduced to principal components (PCs) that explain the maximal amount of variation within a sample. To gain an extensive comparison of the metabolic profiles of the three albescent stages (YG, WI and WII) and the G stage, a PCA (four components, R^2^X = 0.802, Q^2^ = 0.302) score plot (Fig 2) was constructed. Variation in the data set that was explained by one PC (PC1), two PCs (PC1 and PC2), three PCs (PC1, PC2 and PC3) and four PCs (PC1, PC2, PC3 and PC4) was 44.39%, 62.76%, 72.46% and 80.02%, respectively. QC samples were grouped together, indicating that the QC samples had similar metabolic profiles and that our entire analysis had satisfactory stability and repeatability. A large variation in water content between groups can cause an over-representation of the sample groups with lower water content due to the use of fresh mass to prepare the QC. This variation likely led to the QC sample being closer to WI and WII than the other groups.

From the PCA analysis results, the samples from the three albescent stages (YG, WI and WII) and the G stage were grouped into four distinct areas, indicating that the samples within distinct stages had similar metabolic profiles. Overlap between the WI and WII stages was identified, suggesting that parts of their metabolic profiles were similar. This plot suggested that the differences in metabolic profiles detected in the data set were likely correlated to the different color stages during new shoot development.

### Differential metabolites of the three albescent stages (YG, WI and WII) and the G stage based on partial least squares discriminant analysis (PLS-DA)

**Fig 3:**
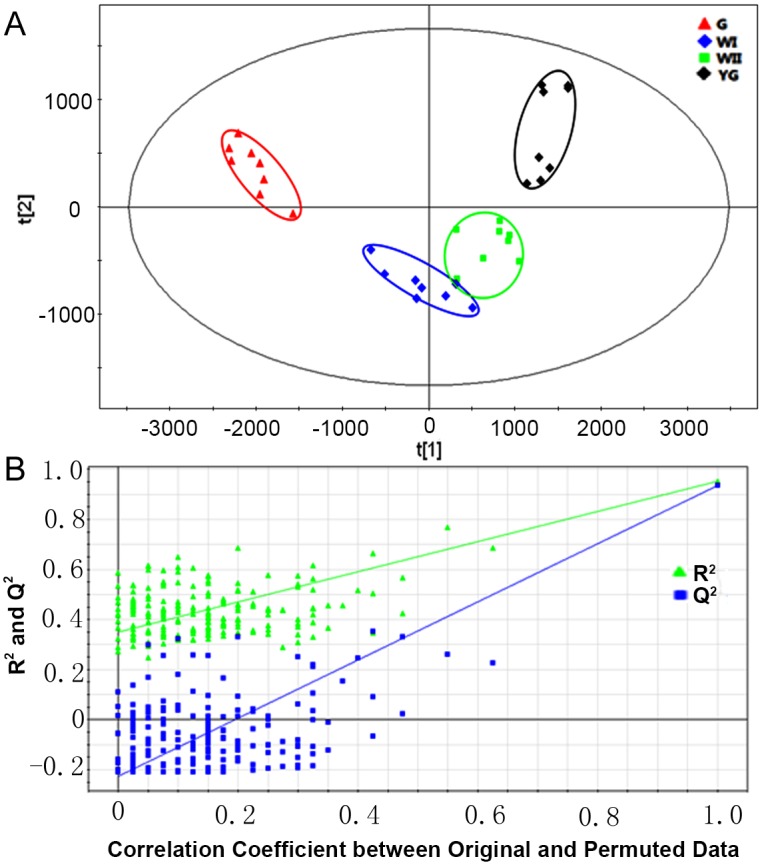
PLS-DA score scatter plots and permutation tests. (A) PLS-DA score plots resulted from the metabolites in tea plant leaves in the three different albescent stages (YG, WI and WII) and the G stage. The sampling groups are color coded as follows: black = YG-stage leaves; blue = WI-stage leaves; green = WII-stage leaves; and red = G-stage leaves. (B) Permutation tests of the PLS-DA model indicate the absence of over-fitting in the model.

PLS-DA is a multivariate classification method based on PLS, which is the regression extension of PCA. PLS can also be used in discriminant analysis and is referred to as PLS-DA. PLS-DA explains the maximum separation between defined class samples. To investigate the differential metabolites among the three different albescent stages (YG, WI and WII) and the G stage, PLS-DA was applied to the 32 samples from the YG, WI, WII and G stages. PLS-DA analysis of the GC–MS data resulted in a model with R^2^X = 0.517, R^2^Y = 0.952, and Q^2^ = 0.935, indicating a good fit and high prediction ability (Fig 3A). Clear differences were shown in the PLS-DA score plots obtained from the tea plant GC–MS data among the YG stage, the two white stages (WI and WII stages) and the G stage. This result indicated that the metabolic profiles differed between the various albescent stages (YG, WI and WII) and the G stage. A chance permutation test (R^2^Y-intercept = 0.35, and Q^2^-intercept = –0.226) showed an absence of over-fitting (Fig 3B). Additionally, no obvious separation trend was detected between the WI and WII stages, suggesting that parts of their metabolic profiles were similar.

**Fig 4:**
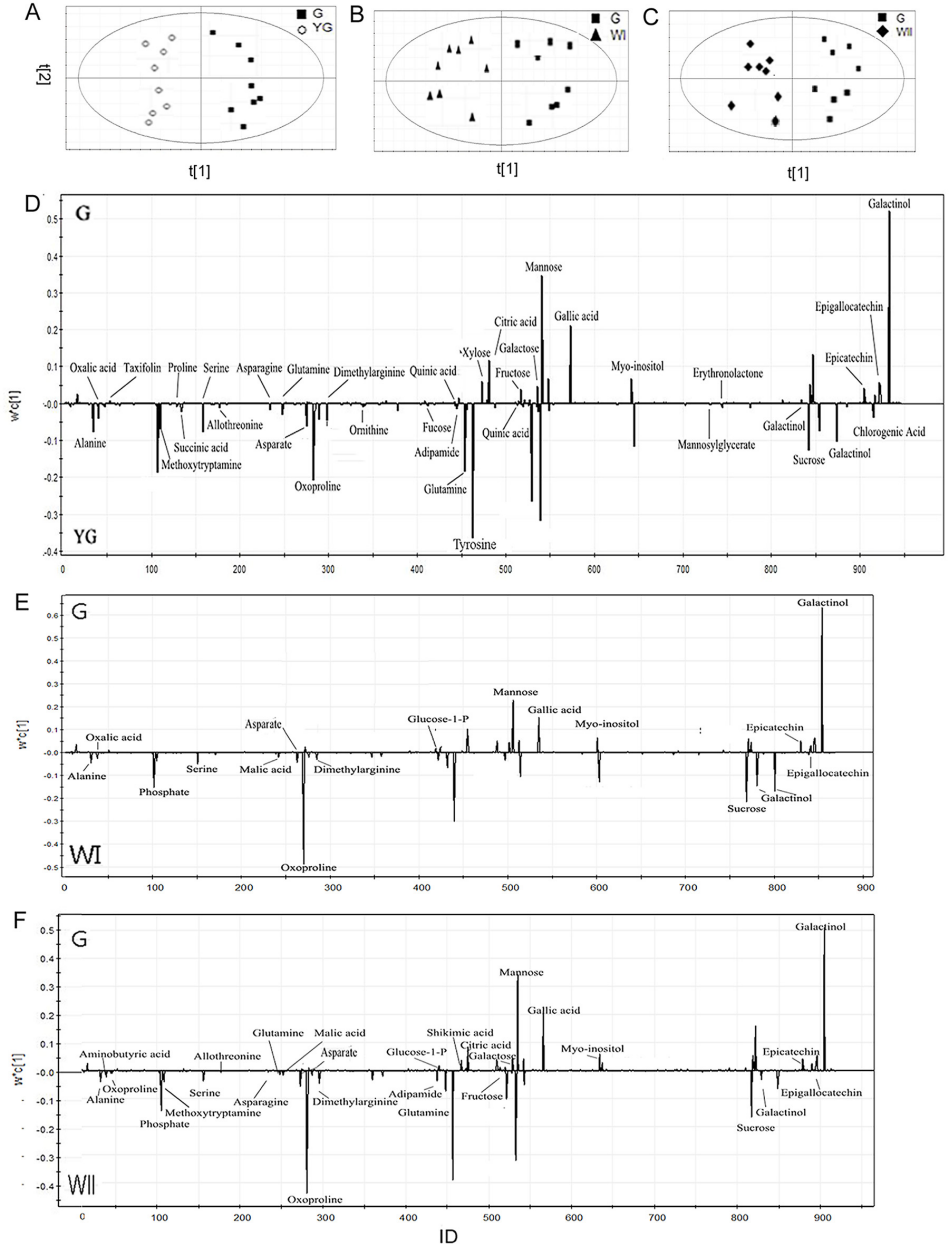
PLS-DA model plots and loading plots for the YG, WI and WII stages versus the G stage. Score plots of pairwise comparisons between the (A) YG stage versus G stage, (B) WI stage versus G stage and (C) WII stage versus G stage. Loading plots of the pairwise comparisons between the (D) YG stage versus G stage, (E) WI stage versus G stage and (F) WII stage versus G stage.

To identify the metabolite contributions of each albescent stage (YG, WI and WII) that are distinct from those of the G stage, PLS-DA models were used to generate pairwise comparisons of the YG, WI and WII stages with the G stage. All PLS-DA models showed a high predictability (Q^2^) and strong goodness of fit (R^2^X), calculated as 0.98 and 0.64 in comparing the YG and G stages, 0.91 and 0.52 in comparing the WI and G stages, and 0.95 and 0.57 in comparing the WII and G stages, respectively. Finally, all the models were validated using a permutation test. In the PLS-DA models, the YG, WI and WII stages were clearly separated from the G stage, suggesting a marked difference in the metabolic profiles between each of the three albescent stages (YG, WI and WII) and the G stage (Fig 4A, 4B and 4C). A metabolite with a VIP value greater than 1 could be considered as a significant metabolite for the separation of sample groups within PLS-DA models [26]. With a cutoff VIP value (VIP > 1) and Student's t test or Kruskal-Wallis test (*p* < 0.05), the differential metabolites in the YG, WI and WII stages compared with the G stage were identified (S4 Table). These metabolites included amino acids, sugars, fatty acids, organic acids and their derivatives.

The major metabolic perturbation causing the differences between the YG, WI and WII stages and the G stage were obtained with line plots of the *X*-loadings of the first component in the PLS-DA models (Fig 4D, 4E and 4F). All the metabolites with negative values were at higher concentrations in the stages with negative scores (the YG, WI and WII stages), and all the metabolites with a positive value were present at higher concentrations in the stage with positive scores (the G stage) (Fig 4D, 4E and 4F). The corresponding loading was negative at the positions of free amino acids (alanine, serine and aspartate) and sugar (sucrose) during the YG, WI and WII stages, and positive at the positions of organic acids (gallic acid, epicatechin and epigallocatechin) during the G stage (Fig 4D, 4E and 4F). A previous study found that the sucrose content increases during tea shoot development [28]. In the present study, the sucrose concentration was higher in the YG, WI and WII stages and lower in the G stage. With the development of the leaves from the YG stage to G stage, the leaf size becomes much larger in the G stage than in the YG, WI and WII stages. Although the sucrose concentration was low in the G stage, the sucrose content might appear higher because of the larger leaf size. In our study, higher amino acid and lower epicatechin and epigallocatechin concentrations were found in the three different albescent stages (YG, WI and WII) compared with the G stage (Fig 4D, 4E and 4F).

**Fig 5:**
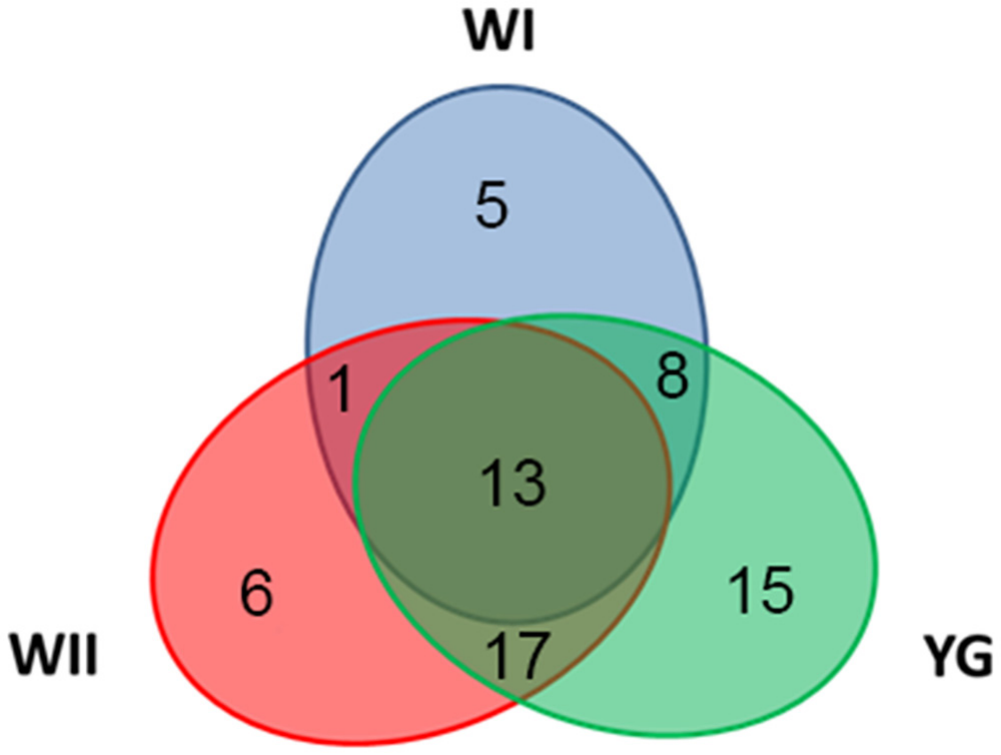
Venn diagram of the differential metabolites from the three albescent stages (YG, WI and WII) compared with the G stage. The Venn diagram shows the overlapping and stage-specific differential metabolites from the three stages: the YG stage, WI stage and WII stage. The three stages shared 13 differential metabolites compared with the G stage. Some stages demonstrated stage-specific differential metabolites (YG 15, WI 5, WII 6), whereas others overlapped during two stages.

Differential metabolites during the various albescent stages (YG, WI and WII) were compared using a Venn diagram (Fig 5 and S5 Table). The three different albescent stages (YG, WI and WII) shared 13 differential metabolites, mainly comprising sugars and amino acids. These metabolites largely contribute to the differences in metabolic profiles among the three different albescent stages (YG, WI and WII) and the G stage. The number of differential metabolites found only in each albescent stage was 15 for the YG stage, 5 for the WI stage and 6 for the WII stage. These metabolites were considered as the defining metabolites for each albescent stage.

### Differential metabolic pathways among the three albescent stages (YG, WI and WII) and the G stage

**Fig 6:**
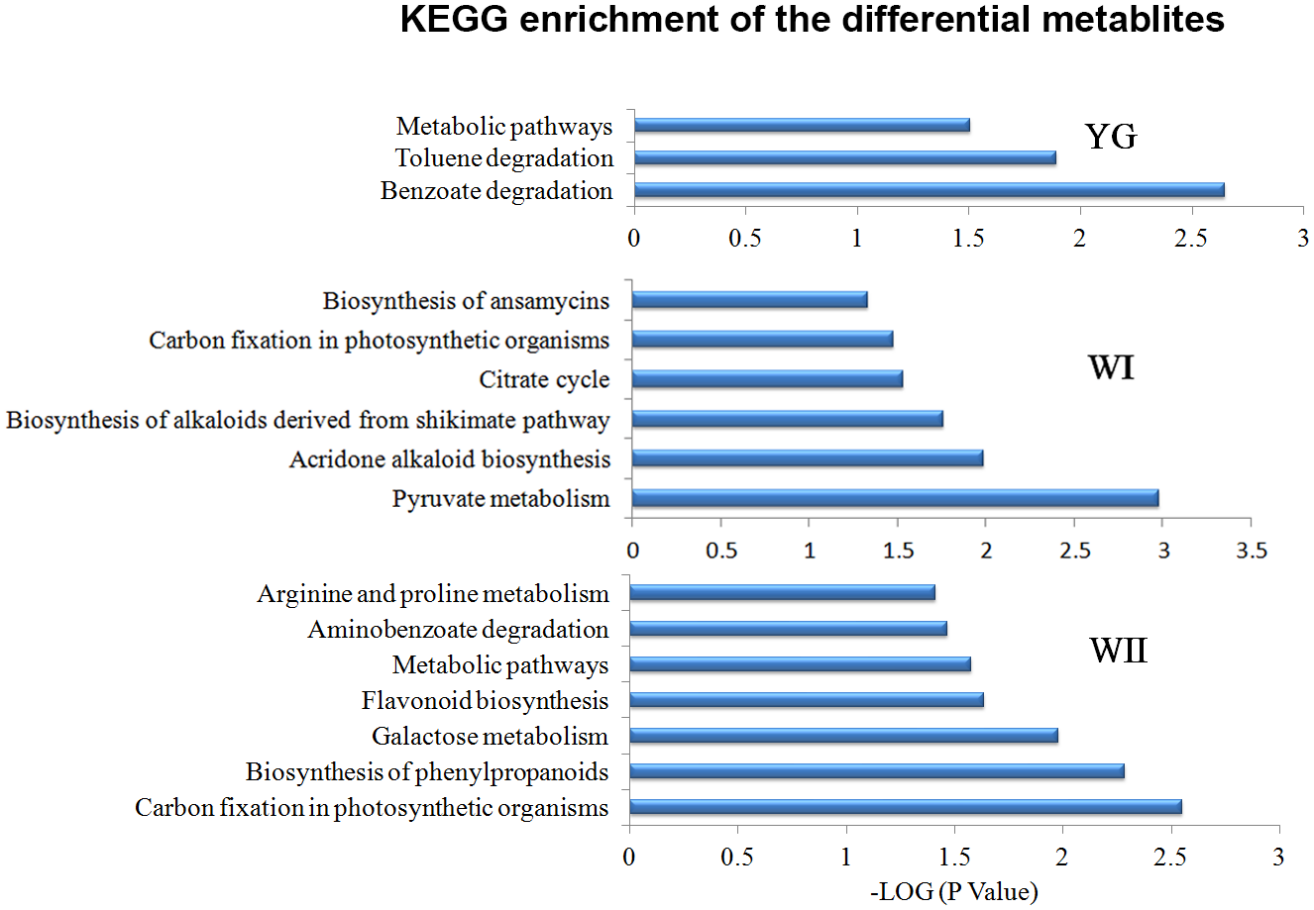
Enrichment of the differential metabolites to distinct KEGG pathways. Differential metabolites in the YG, WI, WII stages compared with those in the G stage were mapped to the distinct metabolic pathways. Enrichment p-values were computed from a hypergeometric distribution. A p-value cutoff of 0.01 was selected to reduce the false discovery rate.

To gain insight into the different metabolic pathways of the albescent stages, the differential metabolites were mapped to the KEGG database (http://www.genome.jp/kegg/) and retained for detailed pathway information (Fig 6 and S6 Table). The differential metabolites of the YG stage were mainly involved in metabolic and toluene- and benzoate-degradation pathways. The differential metabolites in the WI stage were mainly involved in carbon fixation in photosynthetic organisms, in the biosynthesis of alkaloids derived from the shikimate pathway, in the citrate cycle, in acridone alkaloid biosynthesis, in pyruvate metabolism and in the biosynthesis of ansamycins. The differential metabolites in the WII stage were mainly involved in carbon fixation in photosynthetic organisms, in the biosynthesis of phenylpropanoid, in galactose metabolism, in flavonoid biosynthesis, and in arginine and proline metabolism. Carbon fixation in photosynthetic organisms was a shared differential metabolic pathway for the WI and WII stages. A strong relationship was found between the proportion of light absorbed by chlorophyll II and the efficiency of carbon fixation in photosynthetic organisms [29]. 'Anji Baicha' leaves in the white stages (WI and WII stages) contained less chlorophyll II (Fig 1B), and the efficiency of carbon fixation was influenced compared with that of the G stage. Flavonoids are a group of plant polyphenol secondary metabolites that contribute many key characteristics of the tea plant. These compounds are synthesized in the general phenylpropanoid pathway. In the WII stage, the phenylpropanoid and flavonoid biosynthesis pathways differed from those in the G stage. Previous research found that the expression of genes involved in the flavonoid biosynthetic pathway was affected by albescence [4]. The changes observed in the flavonoid biosynthetic pathway may result in altered flavonoid content such as decreased catechins during the albescent stages of the plant.

Differential metabolites were quantitatively analyzed in the leaves of the YG, WI, WII and G stages (Table 2). The metabolite concentrations of the YG, WI and WII stages were compared with those for the G stage, and the p values were shown in S7 Table. By GC–TOF-MS analysis, the concentration of fructose was 157±28.11 in the YG stage and increased to 357±22.53 in the G stage. Fructose concentration significantly decreased (P < 0.05) in the YG, WI and WII stages compared with that in the G stage. By UPLC–QqQ-MS analysis, the concentrations of alanine, glycine, serine, citrulline, glutamine, proline, and valine were 14.3±1.46, 13.57±3.27, 49.06±4.30, 1.32±0.32, 155.81±18.1, 3.07±0.48 and 4.74±1.97 in the YG stage and decreased to 1.87±0.86, 1.36±0.35, 10.27±0.96, 0.27±0.03, 2.34±0.49, 1.24±0.18 and 1.1±0.32 in the G stage. The concentration of tryptophan was 23.2±2.54 in the WI stage and decreased to 4.39±1.09 in the G stage. These metabolite concentrations significantly increased in the YG, WI and WII stages (P < 0.05) compared to those in the G stage. The concentrations of glucose-1-phosphate and epicatechin were 9.82±0.55 and 78.19±8.61 in the WII stage and increased to 47.7±5.86 and 163.66±16.26 in the G stage. The glucose-1-phosphate and epicatechin concentrations significantly decreased (P < 0.05) in the YG, WI and WII stages compared to those in the G stage. These results were consistent with the metabolomic results, which indicated that the differential metabolites identified in our study truly reflected the actual different metabolic profiles in the YG, WI and WII stages compared with those in the G stage.

**Table 2:** Quantification analyzed differential metabolites in the YG, WI, WII stages and the G stage.

Compound (mg/g)	YG	WI	WII	G
Citrulline ^*L*^	1.32±0.32**	0.33±0.02*	0.76±0.07**	0.27±0.03
Epicatechin ^*L*^	150.83±22.40*	102.06±11.81**	78.19±8.61*	163.66±16.26
Glucose-1- phosphate ^*L*^	40.17±6.21**	33.79±6.63**	9.82±0.55**	47.7±5.86
Glutamine ^*L*^	155.81±18.11**	64.37±2.24**	99.56±14.82**	2.34±0.49
Glycine ^*L*^	13.57±3.27**	8.34±0.82**	10.53±1.06**	1.36±0.35
L-Alanine ^*L*^	14.3±1.46**	6.74±0.72**	11.04±1.58**	1.87±0.86
Proline ^*L*^	3.07±0.48**	2.39±0.67**	2.6±0.26**	1.24±0.18
Serine ^*L*^	49.06±4.30**	40.42±5.06**	31.8±4.34**	10.27±0.96
Tryptophan ^*L*^	19.65±1.13**	23.2±2.54**	17.93±1.90**	4.39±1.09
Valine ^*L*^	4.74±1.97**	3.24±1.89**	3.87±0.80**	1.1±0.32
Fructose ^*G*^	260.4±37.87*	157±28.11*	224.7±18.71*	357±22.53

Data are presented as mean±SD. *G* was analyzed by GC–TOF-MS, and *L* was analyzed by UPLC–QqQ-MS.

* indicated P < 0.05 with Student's t test compared with the G stage,

** indicated P < 0.01 with Student's t test compared with the G stage.

### Albescence in the tea plant induces metabolic reprogramming that involves multiple metabolic pathways

**Fig 7:**
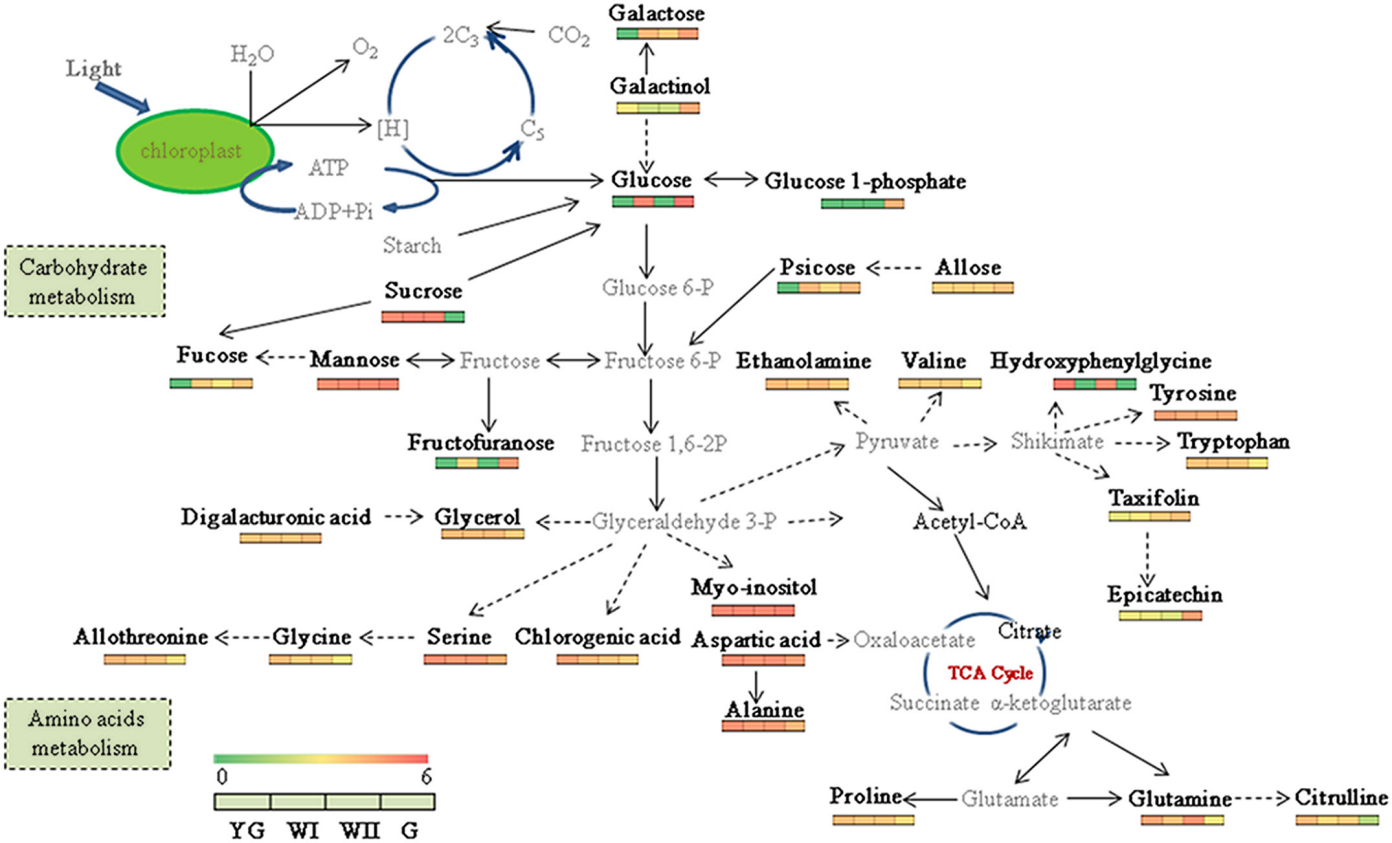
Differential metabolite contents in the YG, WI, WII and G stages of the tea plant. Differential metabolites identified in the distinct albescent stages compared with the G stage in this study are shown in bold. Red denotes the highest concentration, and green denotes the lowest concentration. The scale (ranging from 0–6) represents the log 10 (average value in each stage +1) of the differential metabolites in the YG, WI and WII stages compared with those in the G stage.

Metabolic changes were observed during the YG, WI and WII stages compared with the G stage. The differential metabolites were mainly involved in carbohydrate and amino acid metabolism. The heat maps illustrate the concentrations of these differential metabolites in the YG, WI, WII and G stages using a log ratio (Fig 7 and S8 Table).

Plants use carbon dioxide to synthesize the sugars required for energy, development and survival. Our results showed that the carbon fixation in the photosynthetic organisms’ pathway differed during the WI and WII stages compared with the G stage (Fig 6). The primary products from carbon fixation during photosynthesis are sugars, which are ubiquitous and critical components for general metabolism. These metabolites affect most, if not all, processes in plant cells by providing a carbon skeleton for organic compounds and by storing energy for chemical reactions. Sugars also serve as critical signaling molecules both for cellular metabolism and for biotic and abiotic stress responses [30,31]. In the present study, the differential metabolites during the albescent stages included numerous sugars, such as mannose, galactose and fructofuranose. Glucose was more abundant during the YG and WII stages than during the WI and G stages (Fig 7). Glucose is a general nutrient used by most organisms and plays important roles in carbon storage, biosynthesis, energy supply, and carbon-skeleton construction. Glucose is also a fundamental regulator of many important biological processes, including root, stem, and shoot growth, seedling development, carbon and nitrogen metabolism, photosynthesis, senescence and stress responses [32]. This sugar is interconnected with multiple other pathways and thus might affect the overall reaction networks [22,24]. To meet the demand for carbohydrates during development, woody plants accumulate carbohydrates during periods of excess production and deplete carbohydrates when the rate of utilization exceeds the rate of production [33]. A larger proportion of fixed carbohydrates are retained as polysaccharides such as starches, which provides a larger store to support metabolism and the export of carbohydrates. In our study, we found that glucose was more abundant in the WI stage despite photosynthesis being reduced in this stage; this increase in glucose may be due to increased polysaccharide hydrolysis to meet the needs of the plant in this stage. The glucose-1-phosphate and fructofuranose concentrations were lower during the YG, WI and WII stages than in the G stage. The biosynthesis and metabolism of these metabolites may be largely influenced by albescence of the tea plant. The myo-inositol, mannose, and allose concentrations did not significantly change during the three albescent stages (YG, WI and WII) and the G stage (Fig 7).

The digalacturonic acid and ethanolamine concentrations were maintained at high concentrations during the three albescent stages (YG, WI and WII) and the G stage (Fig 7). The glycerol concentrations was higher during the YG, WI and WII stages than its in the G stage. The taxifolin concentration continually increased with the development of leaves from the YG stage to the G stage. The epicatechin concentrations were similar during all three albescent stages (YG, WI and WII) but increased during the G stage (Fig 7). Lin *et al*. reported a higher catechin concentration in older leaves [34]. This finding is consistent with our results, which showed that older leaves from the G stage contained more epicatechin than younger albino leaves.

Lin *et al*. also indicated that the inhibition of chlorophyll biosynthesis led to a shift toward amino acid biosynthesis during the albescent stages of 'Anji Baicha' [2]. In our study, the majority of amino acids, including allothreonine, glycine, serine, tryptophan, citrulline, glutamine, alanine, valine and proline, increased during the albescent stages compared with the re-greening stage (Fig 7). Free amino acids such as theanine, glutamine, and serine are key components of the umami flavor in green tea made from albino cultivars [2]. Glutamine is the direct substrate for theanine biosynthesis, and increased glutamine contents can enhance theanine biosynthesis in tea seedlings [35]. Thus, increased glutamine concentrations during the albescent stages may contribute to the high contents of theanine in 'Anji Baicha'. Glutamine also serves in the C5 pathway as the direct precursor of alanine [36], the concentration of which was also increased during the albescent stages. Proline could be synthesized in two pathways (glutamate and ornithine pathways). Increased proline content resulted from the coordinated inhibition of proline degradation and activation of proline biosynthesis. In our study, proline concentrations were much higher during the albescent stages than during the re-greening stage. Thus, proline accumulation might be the result of the inhibition of its degradation and activation of its biosynthesis in the 'Anji Baicha' plant during the early spring.

## Conclusion

This work applied a GC–MS-based metabolomics approach to study the metabolic profile changes during the various albescent stages and the re-greening stage. Our results reveal that metabolic profiling could effectively discriminate among the albescent stages and the re-greening stage. Albescence accompanied by the development of new shoots mainly disturbed the metabolites involved in the citrate cycle, carbon fixation in photosynthetic organisms and phenylpropanoid biosynthetic metabolic pathways. These changes resulted in changes in the concentrations of metabolites involved in carbohydrate and amino acid metabolism. During the albescent stages, characterized by lower chlorophyll concentration, photosynthesis was inhibited, and organic acid and amino acid concentrations were changed. The results obtained in this study improved our current understanding of the metabolic mechanisms involved during albescence in the tea plant.

## Supporting Information

S1 Table The validation information of several differential metabolites by reference standards. (DOC)

S2 Table All the metabolites identified in the various stages of tea leaves. (XLSX)

S3 Table The data used for PCA and PLS-DA analysis. (XLSX)

S4 Table Differential metabolites identified in the YG, WI and WII stages compared with the G stage. (XLSX)

S5 Table List of the metabolites that fit into each section of the Venn diagram. (XLSX)

S6 Table Differential metabolites involved in different metabolic pathways by KEGG database enrichment. (XLSX)

S7 Table p-Values for the quantification analysis of differential metabolites in YG, WI, WII stages compared with the G stage. (DOC)

S8 Table Differential metabolite contents in the YG, WI, WII and G stages of the tea plant. (XLSX)
