# Supplementary material for: Differential Metabolic Profiles during the Albescent Stages of ‘Anji Baicha’ (Camellia sinensis)
Source: PLoS One. 2015 Oct 7;10(10):e0139996. doi: 10.1371/journal.pone.0139996 (PMC4622044; doi:10.1371/journal.pone.0139996)
Supplement: S1 Table — (DOC) [file pone.0139996.s001.doc]

**S1 Table. The validation information of some differential metabolites by reference standards**

| Metabolite | Retention time (min) | Typical fragment ions |
| --- | --- | --- |
| Fructose | 28.83 | 147, 299, 315, 438, 589 |
| Valine | 10.08 | 144, 218 |
| Proline | 11.35 | 142, 216 |
| Glycine | 11.43 | 147, 174, 248 |
| Serine | 12.08 | 147, 204, 218 |
| Glutamine | 14.06 | 147, 155, 245 |
| Tryptophan | 24.29 | 202, 218, 291 |
| Citrulline | 20.20 | 157, 188 |
| Epicatechin | 32.92 | 123, 139, 152, 368, 290 |
