# Supplementary material for: Differential Metabolic Profiles during the Albescent Stages of ‘Anji Baicha’ (Camellia sinensis)
Source: PLoS One. 2015 Oct 7;10(10):e0139996. doi: 10.1371/journal.pone.0139996 (PMC4622044; doi:10.1371/journal.pone.0139996)
Supplement: S7 Table — (DOC) [file pone.0139996.s007.doc]

**S7 Table. p-Values for the quantification analysis of differential metabolites in YG, WI, WII stages compared with the G stage**

| Metabolite | YG | WI | WII |
| --- | --- | --- | --- |
| Citrulline *^L^* | 0.005 | 0.039 | 0.005 |
| Epicatechin *^L^* | 0.037 | 0.008 | 0.047 |
| Glucose-1- phosphate *^L^* | 0 | 0 | 0 |
| Glutamine *^L^* | 0 | 0 | 0 |
| Glycine *^L^* | 0 | 0.004 | 0 |
| L-Alanine *^L^* | 0.007 | 0.008 | 0 |
| Proline *^L^* | 0 | 0.003 | 0 |
| Serine *^L^* | 0 | 0.002 | 0 |
| Tryptophan *^L^* | 0.002 | 0 | 0 |
| Valine *^L^* | 0 | 0.004 | 0.005 |
| Fructose *^G^* | 0.026 | 0.038 | 0.034 |
